# Antibacterial mechanism of areca nut essential oils against *Streptococcus mutans* by targeting the biofilm and the cell membrane

**DOI:** 10.3389/fcimb.2023.1140689

**Published:** 2023-08-28

**Authors:** Shuwei Liu, Tiantian Zhang, Zhijin Li, Yan Wang, Lei Liu, Zhenbo Song

**Affiliations:** ^1^National Engineering Laboratory for Druggable Gene and Protein Screening, School of Life Sciences, Northeast Normal University, Changchun, China; ^2^College of Ecology and Environment, Hainan Tropical Ocean University, Sanya, China; ^3^Xiamen Key Laboratory of Natural Medicine Research and Development, Xiamen Health and Medical Big Data Center (Xiamen Medicine Research Institute), Xiamen, China; ^4^NMPA Key Laboratory for Quality Control of Cell and Gene Therapy Medicine Products, Northeast Normal University, Changchun, China

**Keywords:** *Streptococcus mutans*, antibacterial activity, antibiofilm mechanism, essential oil, areca nut

## Abstract

**Introduction:**

Dental caries is one of the most common and costly biofilm-dependent oral diseases in the world. *Streptococcus mutans* is the major cariogenic pathogen of dental caries. *S. mutans* synthesizes extracellular polysaccharides by autologous glucosyltransferases, which then promotes bacterial adhesion and cariogenic biofilm formation. The *S. mutans* biofilm is the principal target for caries treatment. This study was designed to explore the antibacterial activity and mechanisms of areca nut essential oil (ANEO) against *S. mutans*.

**Methods:**

The ANEOs were separated by negative pressure hydro-distillation. The Kirby-Bauer method and broth microdilution method were carried out to evaluate the antibacterial activity of different ANEOs. The antibacterial mechanism was revealed by crystal violet staining, XTT reduction, microbial adhesion to hydrocarbon test, extracellular polysaccharide production assay, glucosyltransferase activity assay, lactate dehydrogenase leaking, propidium iodide staining and scanning electron microscopy (SEM). The cytotoxicity of ANEOs was determine by MTT assay.

**Results:**

The ANEOs separated at different temperatures exhibited different levels of antibacterial activity against *S. mutans*, and the ANEO separated at 70°C showed the most prominent bacteriostatic activity. Anti-biofilm experiments showed that the ANEOs attenuated the adhesion ability of *S. mutans* by decreasing the surface hydrophobicity of the bacteria, prevented *S. mutans* biofilm formation by inhibiting glucosyltransferase activity, reducing extracellular polysaccharide synthesis, and reducing the total biofilm biomass and activity. SEM further demonstrated the destructive effects of the ANEOs on the *S. mutans* biofilm. Cell membrane-related experiments indicated that the ANEOs destroyed the integrity of the cell membrane, resulting in the leakage of lactic dehydrogenase and nucleic acids. SEM imaging of *S. mutans* cell showed the disruption of the cellular morphology by the ANEOs. The cytotoxicity assay suggested that ANEO was non-toxic towards normal oral epithelial cells.

**Discussion:**

This study displayed that ANEOs exerted antibacterial activity against *S. mutans* primarily by affecting the biofilm and disrupting the integrity of the cell membrane. ANEOs has the potential to be developed as an antibacterial agent for preventing dental caries. Additionally, a new method for the separation of essential oil components is presented.

## 1 Introduction

Dental caries is a sugar-driven, biofilm-mediated, multifactorial, and dynamic disease driven by an ecological imbalance in the physiological equilibrium between tooth minerals and oral microbial biofilms, and can result in the phasic demineralization (Pitts et al., 2017). The occurrence of dental caries involves various cariogenic and commensal microbes in biofilms on tooth surfaces (Koo et al., 2013; Zhang et al., 2021), and *Streptococcus mutans* is considered as one of the main etiological bacteria (Lemos et al., 2019). *S. mutans* possesses several virulence properties, including adherence to solid surfaces, biofilm formation, production of acid and extracellular polysaccharide (EPS) (Krzyściak et al., 2014; Sun et al., 2023). The EPS is the critical component of the extracellular matrix for cariogenic biofilm formation, which mainly includes water-insoluble polysaccharide (WIP) and water-soluble polysaccharide (WSP) and is synthesized by glucosyltransferases (Gtfs) secreted by *S. mutans* (Bowen and Koo, 2011; Cugini et al., 2019). Therefore, selectively targeting *S. mutans* in dental biofilms has been proposed as an important direction for the prevention and treatment of dental caries (Bowen, 2016).

Despite the use of anticaries agents has made significant progress in the prevention and treatment of dental caries, such as the application of fluoride and chlorhexidine (CHX), there are still serious challenges in controlling of dental biofilms (Wang et al., 2023). Common antimicrobial agents, such as CHX, effectively kill bacterial pathogens in the planktonic state, but have limited antimicrobial efficacy for bacteria in plaque biofilms (Gao et al., 2016). Continuous excessive use of antimicrobial agents would lead to unwanted side effects, for examples xerostomia, hypogeusia, tongue discoloration, exfoliation of the oral mucosa, parotid swelling, abnormal oral sensation, and even serious anaphylaxis (Tartaglia et al., 2019). Hence, novel oral bactericide inhibiting the formation of dental caries biofilms is needed (He et al., 2019). Natural products are potential source of novel antibacterial agents, have broad availability and chemical diversity, and have been used to treat oral infections and diseases (Palombo, 2011). Essential oils are important natural products, showed strong antibacterial activity, and have aroused extensive attention (Freires et al., 2015; Wang et al., 2021). However, the chemical components of essential oils are complex, and it is difficult to separate and enrich the active constituents. Currently, few reports about separation and enrichment methods for active constituents of essential oils exist.

*Areca catechu* L. is a tropical characteristic plant widely distributed in Malaysia, China, India and other Asia countries as well as in the South Pacific islands. The fruit of *Areca catechu*, areca nut, is an important traditional Chinese medicinal material, which has been listed in the Pharmacopoeia of the People’s Republic of China, and is also used as herbal medicines in other countries (Peng et al., 2015). The areca nut is commonly used to treat malaria, ascariasis, food retention, diarrhea, beriberi, arthritis, dental caries and strengthen the teeth (National Administration of Traditional Chinese Medicine Chinese Materia Medica Editorial Committee, 2004; Chen et al., 2021; Ansari et al., 2021). Areca nut contains a variety of bioactive substances including flavonoids, phenolics, tannins, alkaloids and others (Peng et al., 2015), and exhibits many biological activities in modern pharmacology research, such as effect on nervous system (Bhandare et al., 2015), anti-inflammatory effects (Bhandare et al., 2010), and antibacterial and antifungal effects (Machová et al., 2021). Despite areca nut is a well-known traditional herbal medicine in China, but there are not enough reports about the essential oils.

Accordingly, in the present study, we achieved enrichment of areca nut essential oil (ANEO) active components in the laboratory using a tailor-made negative pressure device, investigated the antibacterial activity of ANEOs against *S. mutans*, and analyzed the mechanisms of action of ANEO from the perspectives of biofilm and cell membrane to explore the potential application of ANEO as an oral bactericide.

## 2 Materials and methods

### 2.1 Plant material and bacterial strains

Areca nuts were collected from an areca nut plantation in Sanya, Hainan Province, China. Fresh immature green areca nuts with uniform size were selected and dried to constant weight at 50°C for further use. *S. mutans* (ATCC 25175) was purchased from the Guangdong Microbial Culture Collection Center and cultured in brain heart infusion medium (BHI) supplied by HKM (Guangdong, China).

### 2.2 Preparation of ANEOs

Based on the positive correlation between liquid boiling temperature and air pressure, the ANEOs were extracted in sequence by hydro-distillation under different negative pressures using a tailor-made extraction device (**Figure S1** in the **Supplementary Material**). The dried areca nuts were crushed, passed through a 24-mesh sieve and placed in an extraction flask. The pressure in the essential oil extraction device was adjusted so the extraction solvent boiled at (60 ± 1) °C for 5 h. The areca nut essential oil so obtained was designated as EO-60. The pressure in the device was then adjusted so the extraction solvent boiled at (70 ± 1) °C for 5 h and the essential oil obtained was designated as EO-70. Samples of EO-80, EO-90 and EO-100 were extracted sequentially using this method. The areca nut essential oil extracted by traditional hydro-distillation was designated as EO-Tr. The composition of the ANEOs was analyzed by gas chromatography-mass spectrometry.

### 2.3 Inhibition zone diameter (DIZ) determination

The DIZ was detected by the Kirby–Bauer (K-B) method (CLSI, 2012). *S. mutans* was cultured overnight, and 100 μL of bacterial suspension (1 × 10^7^ CFU/mL) was spread on a BHI agar plate. Sterile filter paper discs (diameter 6 mm) containing 10 μL (or 10 mg, dissolved in Tween 80) of ANEOs were placed on the surface of the agar plate. A paper disc containing 10 μL of 0.6 mg/mL CHX was used as the positive control (James et al., 2017). Tween 80 was the negative control. The inhibition zone diameter was measured after incubating at 37°C for 24 h.

### 2.4 Minimum inhibitory concentration (MIC) determination

The MIC was determined using a broth microdilution method (Chen et al., 2016). The ANEO samples were prepared by a double dilution method using BHI liquid medium in a microplate with final concentrations from 0.015 to 8.0 μL/mL. An equal volume of bacterial suspension (1 × 10^7^ CFU/mL) was added to each well. Tween 80 was used as a cosolvent and the final concentration was 2%. The positive control was CHX and the negative control included 2% Tween 80 but no ANEOs. Twenty microliters of the sterile solution of sodium resazurin (1 mg/mL) were added per well. Finally, the 96-well polystyrene microplates were incubated at 37°C for 24 h. The lowest concentration of ANEOs that prevented the solution from changing from dark blue to pink or light yellow was taken as the MIC value. The above assay was repeated and analyzed at five concentration levels which included the minimum MIC values of the six ANEOs and other four concentration levels.

### 2.5 Minimum bactericidal concentration (MBC) determination

To determine the MBC of each ANEO, 50 μL of suspensions in the above wells at concentrations equal to or higher than the MIC were spread on BHI agar plates and incubated at 37°C for 24 h. The MBC value was the lowest concentration at which no visible colony was observed on the plate.

### 2.6 Construction and treatment of microplate biofilms

According to the literature (Aiassa et al., 2016) with slight modifications, the tested bacterial suspension in the logarithmic phase was adjusted to 1×10^7^ CFU/mL using BHI supplemented with 0.25% sucrose (BHIS). Aliquots of 200 μL of the prepared suspension were added to each well and the plates were incubated at 37°C for 24 h to form biofilms. After incubation, the supernatant was removed and each well was washed three times with sterile PBS. Next, 200 μL of fresh BHI broth with 1/8, 1/4, 1/2 and 1 MIC of each ANEO were added to the wells and the plates were incubated for another 24 h at 37°C. The biofilms were obtained by washing three times with sterile PBS.

### 2.7 Crystal violet assay

Crystal violet was used as an indicator of the total biofilm biomass (Leite et al., 2013). An aliquot of 200 μL methanol was added to the above constructed microplate biofilms to fix the biofilm, and discarded after 15 min. After drying at room temperature, each well was filled with 200 μL of a 1% crystal violet solution and allowed to stand for 5 min. Finally, each well was washed three times with distilled water and a mixture of ethanol and acetone (3:7) was added. The absorbance of each well was measured at 595 nm.

### 2.8 XTT assay

The metabolic activity of the cells in biofilms was evaluated using the XTT [2,3-bis (2-methloxy 4-nitro-5-sulfophenyl)-2H-tetra zolium-5-carbox-anilide] reduction assay (Huang et al., 2012). PBS (100 μL) and 12 μL of an XTT-menadione solution were added into each well prepared according to section 2.6. The XTT-menadione solution was prepared by mixing 25 μL XTT solution (1 mg/mL in PBS) and 2 μL of menadione solution (1 mmol/L in acetone). The absorbance (*A_1_*) was measured at 492 nm after incubating for 3 h in the dark at 37°C. The absorbance of the negative control group was *A_0_*. The biofilm activity was calculated as follows:


$$\mathrm{Biofilm}\;\mathrm{activity}=\frac{{\text{A}}_{1}}{A_{0}}\times 100$$

### 2.9 Determination of bacterial surface hydrophobicity

Bacterial surface hydrophobicity was evaluated by the microbial adherence test with a hydrocarbon (MATH) (Hernández-Alcántara et al., 2018). The biofilms were prepared according to section 2.6 using six-well cell-culture plates. The bacteria were resuspended and collected after two washes with PBS (pH = 7.2). The bacterial suspension was adjusted to an appropriate concentration (OD_560 nm_ = 0.6/0.7, *A_0_*), then mixed with an equal volume of n-hexadecane and stirred for 1 min. The absorbance (*A_1_*) of the water phase was measured at 560 nm after standing for 1 h at 37°C. The hydrophobicity was calculated using the following equation.


$$\mathrm{Hydrophobicity}\left(\%\right)=\frac{A_{0}-A_{1}}{A_{0}}\times 100$$

### 2.10 Extracellular polysaccharide (EPS) quantification

The EPS determination was based on the method of Packiavathy et al. (2012) with a slight refinement. An aliquot of 1.0 mL of bacterial suspension in the log phase and 10 mL of BHIS broth with the ANEOs at a final concentration of MIC were mixed and incubated for 16 h at 37°C. The cultures were centrifuged at 12,000 × g for 30 min at 4°C to collect the supernatant. The sediment was resuspended in sterile water, and centrifuged again. The supernatant collected from both centrifugations was the WSP. The pellet was resuspended in 0.1 mol/L NaOH. The supernatant was collected and filtered through 0.22-µm nitrocellulose membrane filters. The filtered supernatant was precipitated by mixing with three volumes of chilled 95% ethanol and incubating overnight at 4°C to collect the WIP. Negative and positive control groups (CHX) were tested simultaneously.

The EPS were quantified using the phenol/H_2_SO_4_ according to Dubois and co-workers (DuBois et al., 1956). One volume of ice-cold 5% phenol and five volumes of concentrated sulfuric acid were added to one volume of EPS solution. The mixture was incubated at room temperature for 10 min until a red color appeared. The absorbance of the mixture was detected at 490 nm. The inhibition of EPS production was calculated by the following equation.


$$\mathrm{Inhibition}\;of\;\mathrm{EPS}\left(\%\right)=\frac{\mathrm{control}\;OD_{490}-\mathrm{treated}\;OD_{490}}{\mathrm{control}\;OD_{490}}\times 100$$

### 2.11 Gtf activity assay

A crude Gtf extract was prepared by a method previously reported by Koo et al. (2013) with a few modifications. Aliquots of 20 mL of bacterial solutions in log phase were incubated with 200 mL BHI broth containing 1% sucrose at 37 °C for 24 h. The culture was centrifuged at 12000 × g for 30 min at 4°C to collect the supernatant. Ammonium sulfate was added to the supernatant at a final concentration of 60% and the preparation was allowed to stand overnight at 4°C. The precipitate was collected and dialyzed in PBS (pH 6.8) with 1 mM phenylmethyl sulfonyl fluoride (PMSF) as a protease inhibitor for 48 h. The dialyzed solution was the crude extracellular Gtf and was stored at -20°C until use.

The inhibition of Gtf activity by ANEOs was determined by the ability of the Gtf to catalyze the formation of WIP. The method of Ooshima et al. (2000), slightly modified, was used. The reaction mixtures were composed of 1 mL of sterile PBS containing 0.1 M sucrose and 200 µL of prepared crude Gtf. The ANEO samples were added to the reaction mixtures to a final concentration of the MIC and incubated at 37°C for 18 h. Negative and positive control groups (CHX) were run at the same time. The quantity of WIP was determined using the phenol/H_2_SO_4_ method (DuBois et al., 1956). The inhibition of Gtf activity was calculated by the following equation.


$$\mathrm{Inhibition}\;\mathrm{of}\;\mathrm{Gtf}\;activity\left(\%\right)=\frac{\mathrm{control}\;OD_{490}-\mathrm{treated}\;OD_{490}}{\mathrm{control}\;OD_{490}}\times 100$$

### 2.12 Lactate dehydrogenase (LDH) assay

Measurement of LDH leakage was carried out according to Hsu et al. (2021) with slight modifications. A log phase bacterial suspension was adjusted to a concentration of 1×10^7^ CFU/mL and cultured overnight at 37°C. The culture medium was discarded and fresh medium with ANEOs was added to a final concentration of the MIC, then the bacterial cells were incubated for a further 24 h at 37°C. The activity of LDH was measured every 4 h during incubation using a Cytotoxicity LDH Assay Kit-WST (Dojindo, Kumamoto-ken, JPN).

Aliquots of 100 μL of bacterial suspension and 100 μL of an LDH working solution were mixed and incubated in the dark at room temperature for 30 min. Subsequently, 50 μL of stop solution was added to the mixture. The OD_490_ of the bacterial suspension was measured with a multifunctional microplate reader (SpectraMax i3x, Molecular Devices, California, USA). The negative control group was defined as 0% LDH release, and the group treated with 9% Triton X-100 for 24 h was defined as 100% LDH release (Wijesundara et al., 2021).

### 2.13 Nucleic acid leakage assay

Nucleic acid leakage of *S. mutans* treated with ANEOs was detected according to Shen et al. (2015) with minor modifications. A bacterial suspension was centrifuged at 600 × g for 15 min, washed three times with PBS and diluted to 1×10^7^ CFU/mL. The diluted suspension was incubated for 24 h at 37°C with ANEOs at a final concentration of the MIC. Then, 2–3 mL of suspension was removed every 4 h, filtered through 0.22 μm filter membranes, and the absorption was measured at 260 nm.

### 2.14 Propidium iodide (PI) assay

The PI assay was carried out using the method of Souza et al. (2022). The *S. mutans* suspension was diluted to a concentration of 1×10^7^ CFU/mL. Aliquots of 200 μL of bacterial suspension were added to a 96 well plate and incubated at 37°C for 24 h. The supernatant was removed and the wells were washed twice with PBS. A volume of 200 μL fresh BHI was added to the well and the plate was incubated for another 24 h. The biofilms in the well were washed twice with PBS, then covered with 100 μL PBS containing ANEOs at a concentration of MIC and cultured for 12 h at 37°C. After incubation, the biofilms were washed twice with PBS. Finally, the biofilms were covered with 50 µL PI (1 µg/mL), maintained in the dark for 15 min and observed under a fluorescence microscope (RVL-100-G, ECHO, California, USA).

### 2.15 Construction of polystyrene biofilms and scanning electron microscopy (SEM) observation

The experiment was carried out according to Ribeiro-Vidal et al. (2020) with minor modifications. The bottom of a sterile polystyrene Petri dish was trimmed to 3 × 3 mm slices, which were placed in a 24-well cell culture plate. A volume of 100 μL of bacterial culture (1×10^7^ CFU/mL) and 900 μL of BHI broth containing ANEOs were added to the wells. The final concentration of ANEOs was 1/4 MIC. The plate was incubated for 168 h and the culture solution was replaced every 24 h. Then the culture solution was discarded and the polystyrene slices were gently washed with saline. The treated slices were fixed in a 2.5% glutaraldehyde solution for 1 h at 4°C and washed twice with PBS. Next, the slices were dehydrated sequentially in 50%, 70%, 90%, 100% ethanol (dehydrated twice), equal volume mixtures of anhydrous ethanol and tert-butanol, and tert-butanol, each for 15 min. Finally, the slice surfaces were coated with gold after freeze-drying for 4 h and observed using a SEM (JSM-7610F PLUS, JEOL, Tokyo, JPN).

### 2.16 SEM observation

The SEM was used to observe the morphology and structure of *S. mutans* (Bajpai et al., 2013). A log phase bacterial suspension was centrifuged (600 × g, 15 min) and resuspended in PBS for three times. Then the suspension was mixed with ANEOs at the MIC and cultured at 37°C for 12 h. The bacterial cells were obtained by centrifugation and fixed in a 2.5% glutaraldehyde solution overnight at 4°C. Next, the bacterial cells were dehydrated successively using 30%, 50%, 70%, 90% and 100% ethanol and replaced by tertiary butyl alcohol. The dehydrated samples were freeze-dried for 4 h, then sputtered with gold and observed.

### 2.17 Cytotoxicity assay

Cell viability was tested using the MTT method (Patel et al., 2004). Briefly, the human oral epithelial cells were seeded at a density of 1.0×10^4^ cells per well in Dulbecco’s modified Eagle’s medium supplemented with 10% FBS and incubated for 24 h in a humidified atmosphere containing 5% CO_2_ at 37°C. The cells were then incubated with the ANEOs (0.005, 0.05, 0.5 and 5 mg/mL) for 48 h. Next, 20 µL of MTT solution were added to each well and incubated for 4 h. After incubation, the culture medium was removed and an equal volume of DMSO was added to each well. The absorbance was measured at 570 nm. The inhibition rate of cell viability was calculated using the following equation.


$$\mathrm{Inhibition}\;\mathrm{rate}\;\left(\%\right)=\frac{\mathrm{control}\;OD_{570\;}-\mathrm{treated}\;OD_{570}\;}{\mathrm{control}\;OD_{570}}\times 100$$

### 2.18 Statistical analysis

Nine replicates were done for each treatment. The results were expressed as the mean ± standard error. A one-way ANOVA was carried out using the SPSS software version 19.0 (IBM Corp., Armonk, NY, USA). The difference between groups was considered significant when *P*< 0.05. Then, *P*< 0.01 indicated an extremely significant difference and *P* > 0.05 indicated no statistical significance.

## 3 Results

### 3.1 Chemical compositions analysis of different ANEOs

**Figure 1:**
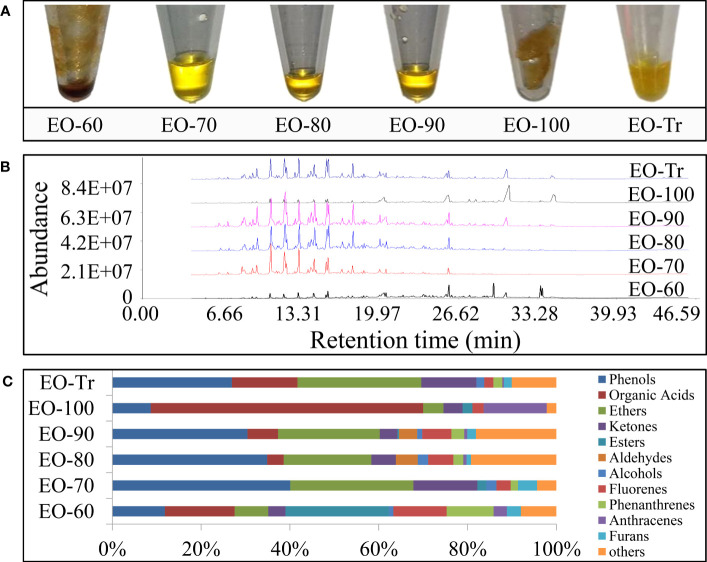
Physical property and chemical composition of separated ANEOs. **(A)** ANEOs. **(B)** GC-MS total ion chromatograms of ANEOs. **(C)** Chemical composition of ANEOs.

The ANEOs extracted under different temperatures are shown in **Figure 1A**. EO-70, EO-80, and EO-90 are liquid, and the color is golden yellow. EO-60, EO-100, and EO-Tr are solid, and appear yellow or dark yellow. In addition, the density of EO-70 is higher than that of distilled water, belonging to heavy oil, while, the density of the other ANEOs is lower, light oil. Total ion chromatograms from ANEOs are seen in **Figure 1B**. The retention times and the abundance of chromatographic peaks indicate that the components of the ANEOs extracted at different temperatures were remarkably different. The similarity of the ANEOs was analyzed with the “Similarity Evaluation System for Chromatographic Fingerprint of Traditional Chinese Medicine 2004A” software (Qian et al., 2020). The similarity between EO-80 and EO-90 is 0.927, and other ANEOs were less than 0.5 (**Table S1** in the **Supplementary Material**).

A total of 75 compounds were identified from the ANEOs by GC–MS analysis, 36 more compounds than that found in EO-Tr (**Table S2** in the **Supplementary Material**). The types of compounds in ANEOs are showed in **Figure 1C**, the proportion of phenolics of EO-70 is the highest (40.09%). Overall, the ANEOs were preliminarily separated through negative pressure hydro-distillation and the phenolics were enriched in EO-70.

### 3.2 Antibacterial activity assessments

**Figure 2:**
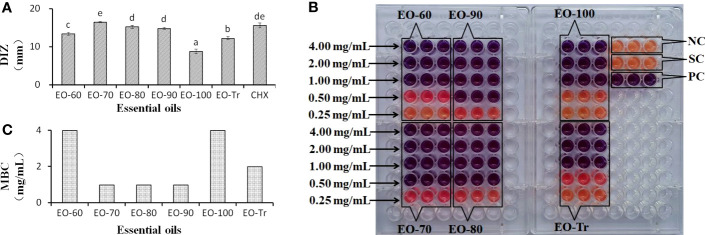
DIZ, MIC and MBC of different ANEOs against *S. mutans*. **(A)** DIZs. **(B)** MICs. **(C)** MBCs. Negative control (NC): BHI + *S. mutans*; Solvent control (SC): BHI + *S. mutans* + Tween 80; Positive control (PC): BHI + *S. mutans* + Tween 80 + CHX; Different lowercase letters indicate significant differences (*P *< 0.05).

The inhibitory activity of the ANEOs on *S. mutans* was assessed by measuring the DIZ, MIC and MBC values (**Figure 2**). All of the ANEOs showed inhibitory effects on *S. mutans*. EO-70 had the largest DIZ value (16.5 ± 0.18 mm), which was greater than that of CHX (15.61 ± 0.68 mm), and clearly inhibited the growth of *S. mutans*. The DIZ of EO-100 was the smallest, and the DIZs of the separated ANEOs except for EO-100 were all significantly larger than that of EO-Tr (*P*< 0.05) (**Figure 2A**). The MIC values of EO-70, EO-80 and EO-90 were 0.50 mg/mL, and those of EO-60, EO-100 and EO-Tr were all 1.0 mg/mL (**Figure 2B**). The MBC values of the ANEOs were shown to be 2 or 4 times of their corresponding MIC values (**Figure 2C**). These results indicate that there are certain differences in the inhibitory effect of different ANEOs against *S. mutans*, and EO-70 exhibits the best antibacterial activity and contains more active substances.

### 3.3 Antibiofilm activity

#### 3.3.1 Effect of ANEO on total biofilm biomass

**Figure 3:**
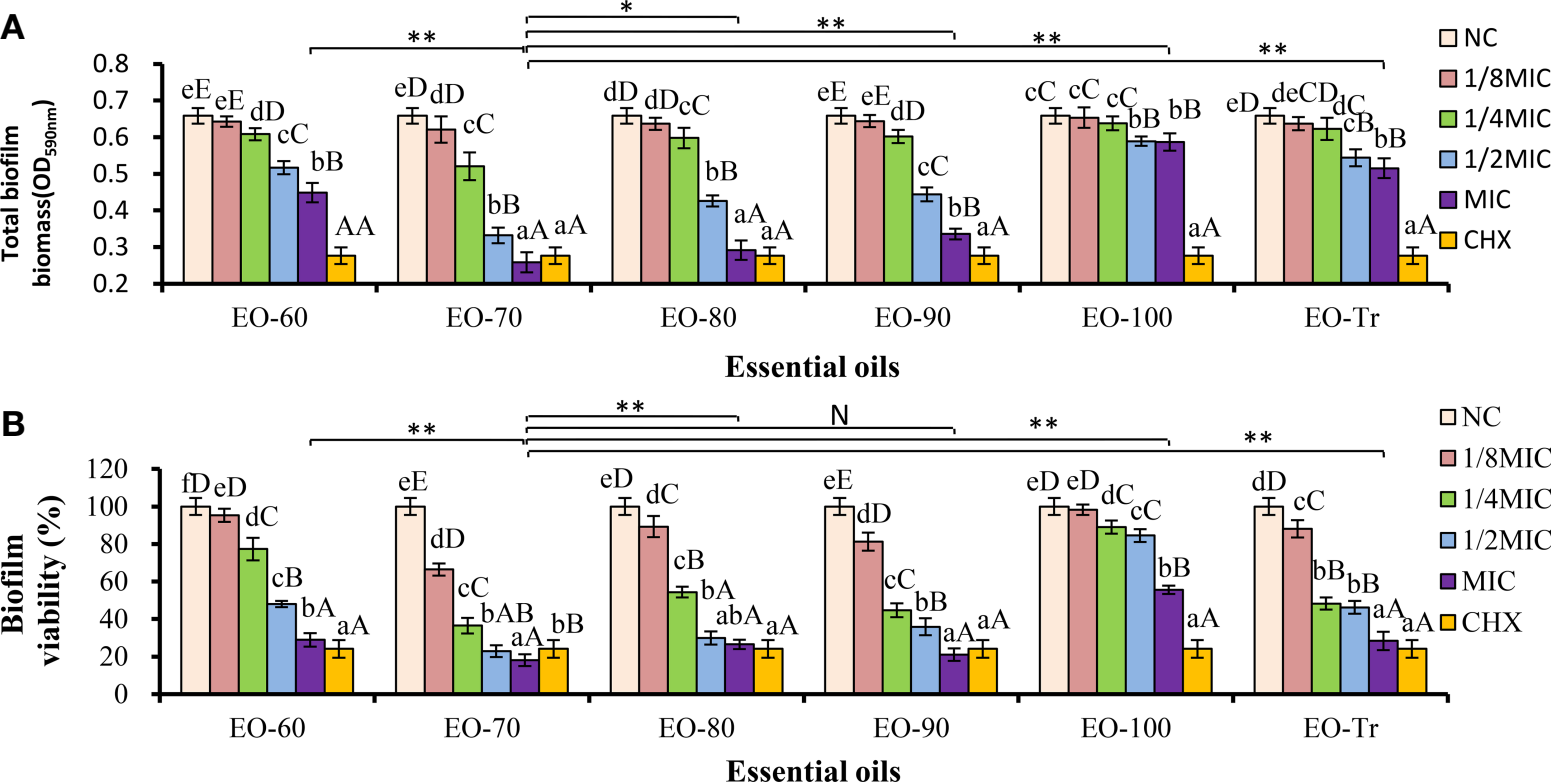
Antibiofilm activity of ANEOs on *S. mutans*. **(A)** Effect of ANEOs on the total biofilm biomass of *S. mutans*. **(B)** Effect of ANEOs on bacterial viability in *S. mutans* biofilms. The NC (or CHX) in the six histograms is the same. Different lowercase letters represent statistically significant differences among the groups of the same ANEO (*P*< 0.05), Different uppercase letters represent statistically extremely significant difference among the groups of the same ANEO (*P *< 0.01). *P < 0.05, **P < 0.01, N represent no statistically significant difference between the groups.

*S. mutans* develops virulence by the biofilm formation on tooth surfaces. Therefore, we firstly examined the effects of different ANEOs on the biofilm biomass. As shown in **Figure 3A**, the total biofilm biomass decreases significantly after treatment with the ANEOs at the MIC concentration compared with NC (*P*< 0.01). Of all the ANEOs at MIC concentration, the total biofilm biomass of EO-70 group is the least, which is significantly different from the other groups (*P<* 0.05). Furthermore, the effect of the ANEOs on the total biofilm biomass is positively correlated with the concentration of ANEOs, and the total biomass of all ANEO groups was the least at the MIC. The change of sample concentrations in the EO-100 and EO-Tr groups has little effect on the total biofilm biomass of *S. mutans*.

#### 3.3.2 Effect of ANEO on biofilm activity

The biofilm activity is represented by the percentage of absorbance value of XTT metabolites in this study. As shown in **Figure 3B**, the biofilm activity of *S. mutans* decreases after culturing with the ANEOs, and the magnitude is positive correlated with the concentration of the ANEOs. At the MIC concentration, the biofilm activity of each ANEO group is extremely significantly less than NC (*P<* 0.01). Furthermore, EO-70 and EO-90 had greater impacts on the biofilm activity than CHX, and still significantly inhibited the bacterial activity at 1/8 MIC. The EO-80, EO-Tr and EO-60 had a moderate inhibitory effect on the biofilm activity, and EO-100 group had the least effect. It can be seen that ANEOs showed a certain inhibitory effect on the biofilm of *S. mutans*.

#### 3.3.3 Effect of ANEO on formation and morphology of biofilm

**Figure 4:**
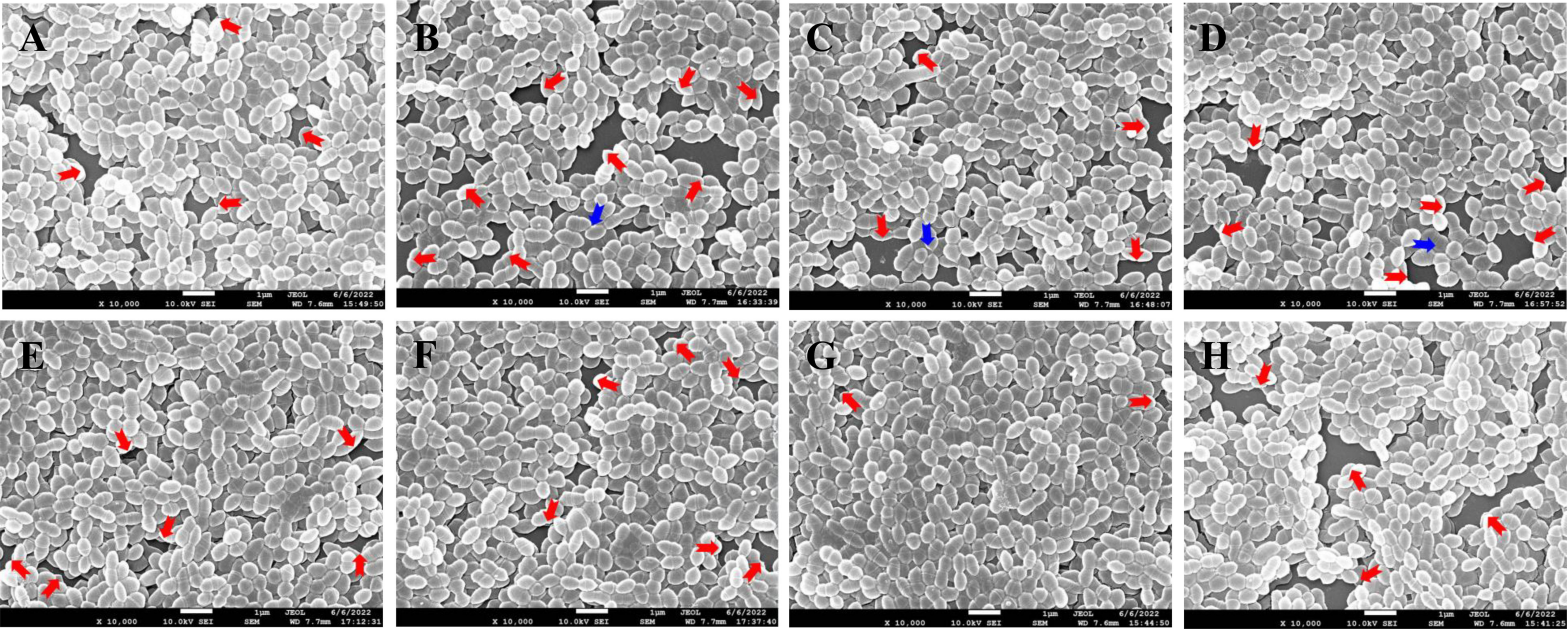
SEM photography of *S. mutans* biofilms treated with different ANEOs (1/4 MIC). **(A)** EO-60, **(B)** EO-70, **(C)** EO-80, **(D)** EO-90, **(E)** EO-100, **(F)** EO-Tr, **(G)** NC, **(H)** CHX. Red arrows point to pores, and blue arrows point to monolayers of cells.

The effect of different ANEOs on the compactness of the cell arrangement in the biofilm was shown in SEM photography. **Figure 4** shows that the compactness of the bacteria was destroyed, the biofilm become thinner, and the bacterial arrangement was not neat but loose. **Figures 4B–D, H** show that the bacterial cell arrangement after treatment with EO-70, EO-80, EO-90, and CHX became sparse with more pores (red arrow), and the biofilms were thin in some places, even with cells in a monolayer (blue arrow). **Figures 4A, E, F** show fewer pores, and the cellular arrangement is relatively dense in the biofilms cultured with EO-60, EO-100 and EO-Tr. The biofilm cells in the NC group show an orderly and compact arrangement with few small pores (**Figure 4G**). This assay demonstrated that the ANEOs could interfere with biofilm formation, affect the cellular arrangement, cause the biofilm to be incomplete, and reduce the biofilm quality, thus reducing the resistance of biofilm to external interference. Simultaneously, the formation of a stable microenvironment becomes difficult, which could reduce the cariogenic capacity of *S. mutans*.

### 3.4 Antibiofilm mechanism

#### 3.4.1 Effect on cell surface hydrophobicity

**Figure 5:**
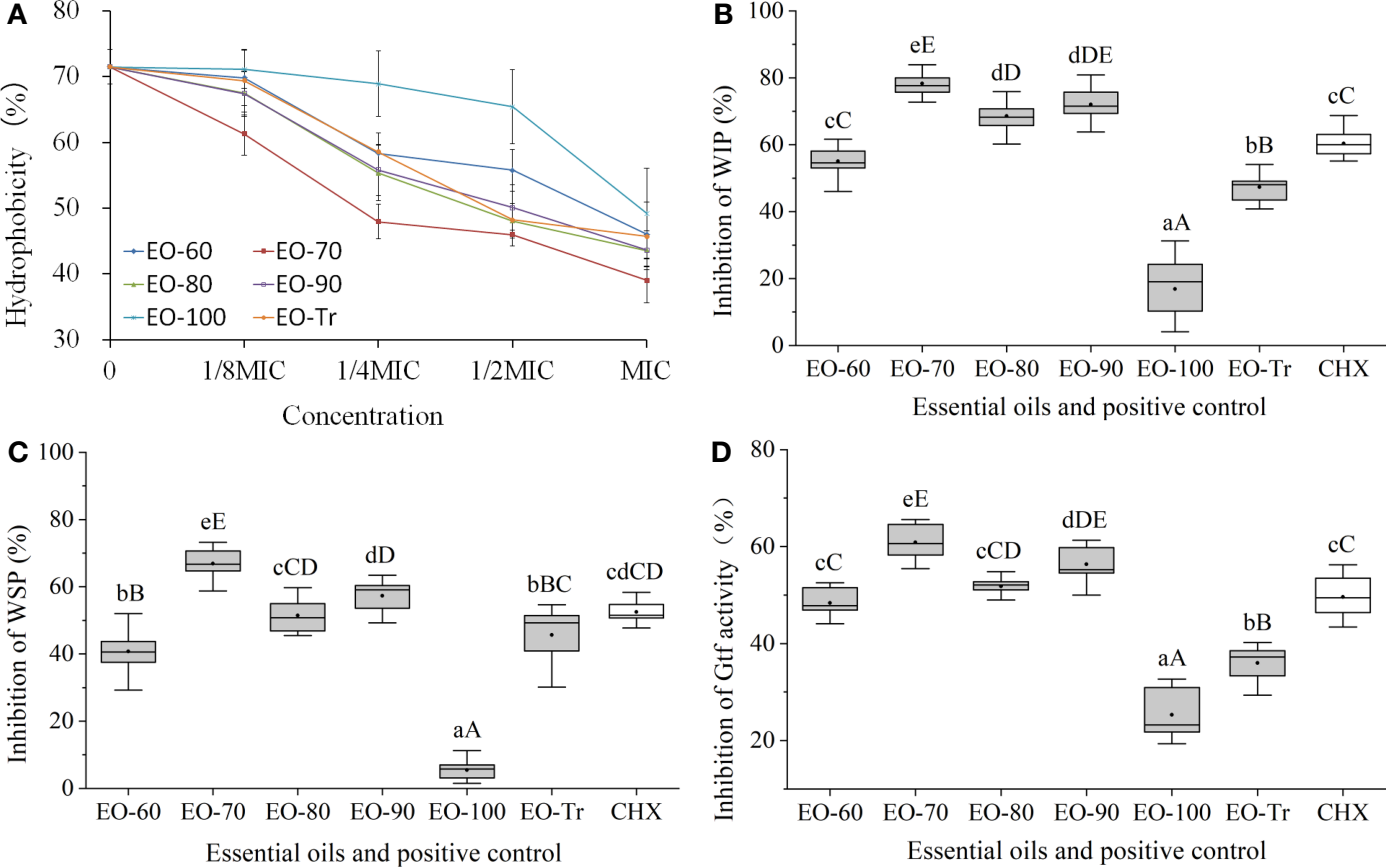
Antibiofilm mechanism of ANEOs. **(A)** Effect of ANEOs on cell surface hydrophobicity. **(B)** Inhibitory effect on the production of WIP. **(C)** Inhibitory effect on the production of WSP. **(D)** Inhibitory effect on Gtf activity. Different lowercase letters represent statistically significant differences among the groups (*P *< 0.05). Different uppercase letters represent statistically extremely significant differences among the groups (*P *< 0.01).

Bacterial cell surface hydrophobicity is closely linked to the bacterial adhesion and biofilm formation, and the higher hydrophobicity rates, the easier of the biofilm formation (Abdulla et al., 2014; Vaca et al., 2020). Therefore, the cell surface hydrophobicity can be used to evaluate whether the ANEOs inhibited the biofilm formation. As shown in **Figure 5A**, the surface hydrophobicity of *S. mutans* decreases with an increase of the ANEOs concentration in the range of 1/8MIC to MIC. The hydrophobicity rate of *S. mutans* treated with EO-70 was the lowest (39.03 ± 3.38%), obviously less than that of the other ANEOs. The result indicated that ANEOs could prevent bacteria from adhering to form biofilms by reducing the surface hydrophobicity of *S. mutans.*

#### 3.4.2 Effects of ANEOs on EPS production

EPS is considered as a virulence factor of cariogenic biofilms (Lin et al., 2021), and included WSP and WIP. The results of the EPS assay showed that the production of WIP and WSP were both inhibited by ANEOs (**Figures 5B, C**). EO-70, EO-80 and EO-90 had good inhibitory effects on the production of WIP, and the inhibition rates were all extremely significantly higher than CHX (*P*< 0.01) (**Figure 5B**). **Figure 5C** shows that the inhibitory effect of EO-70 and EO-90 on the production of WSP is greater than that of CHX, and the difference between EO-70 and CHX is extremely significant (*P<* 0.01). These results indicated that EO-70 and EO-90 had a good inhibitory effect on both WIP and WSP.

#### 3.4.3 Effects of ANEOs on Gtf activity

Gtf is considered to be the key enzyme for the assembly of cariogenic biofilms. Gtf activity can affect the cariogenic potential of *S. mutans* (Ma et al., 2021). **Figure 5D** indicates that all ANEOs exhibit certain inhibition effects on Gtf activity. EO-70 had the greatest inhibitory effects, significantly different than the other five ANEOs and CHX (*P<* 0.05). EO-90 significantly decreased the Gtf activity compared with the other ANEOs (except for EO-70) and CHX (*P*< 0.05). The inhibitory effect of EO-80 was greater than that of CHX but not significantly (*P* > 0.05), and the inhibitory effects of EO-60, EO-100 and EO-Tr was weaker than that of CHX. This illustrated that ANEOs could inhibit Gtf (i.e., EPS synthetase) activity, and the results are consistent with that of the EPS quantification.

### 3.5 Membrane damage studies

#### 3.5.1 Leakage of LDH

**Figure 6:**
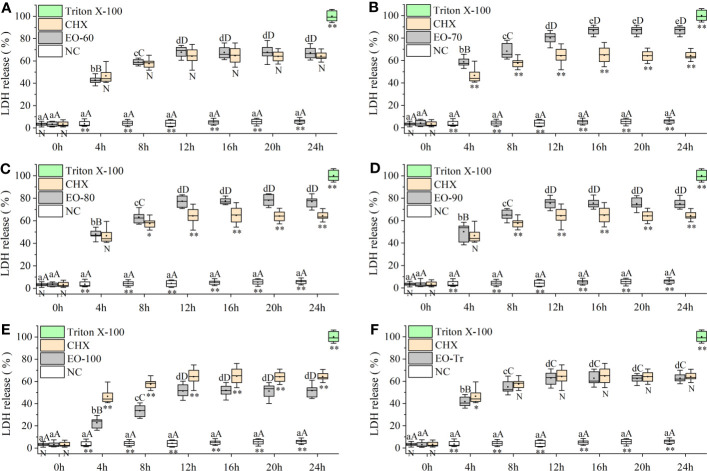
Effect of ANEOs on LDH leakage from *S. mutans*. The concentration of each ANEO is the MIC. **(A)** EO-60, **(B)** EO-70, **(C)** EO-80, **(D)** EO-90, **(E)** EO-100, **(F)** EO-Tr. Different lowercase letters represent statistically significant differences among the experimental time points in the same ANEO group (or NC) (*P* < 0.05) and different uppercase letters represent statistically extremely significant differences (*P* < 0.01). * represents statistically significant differences between the NC (or CHX) and the ANEO groups at the same time point (*P* < 0.05), ** represents statistically extremely significant differences (*P *< 0.01), and N represents no significant difference (*P* > 0.05).

LDH is an important intrinsic enzyme in *S. mutans*, which can catalyze the synthesis of lactic acid from pyruvate. LDH plays a crucial role in bacterial resistance to host innate immunity and biofilm formation. LDH does not leak from normal cells, but is released into the culture medium if the cell membrane is damaged (Yin et al., 2020). LDH release is considered as a reliable indicator of cell membrane damage and permeability increases (Korshed et al., 2018). **Figure 6** shows that, compared with NC, the quantity of released LDH in all the experiment groups is significantly greater (*P*< 0.01) after 4 h, indicating that all ANEOs can damage the cell membrane. The percentage of LDH in the medium of the EO-70 group increased rapidly to 86.59 ± 3.76% within 16 h, and did not increase after 16 h (*P*< 0.05) (**Figure 6B**). **Figures 6A, C–E, F** respectively indicate that the percentage of LDH in the EO-60, EO-80, EO-90, EO-100 and EO-Tr groups show a rapid increase during the first 12 h, to 67.91 ± 4.82%, 77.24 ± 4.98%, 75.41 ± 4.89%, 51.40 ± 5.88% and 62.95 ± 5.95%, and 12 h later the LDH content reached a steady state (*P*< 0.05). These results suggested that ANEOs could destroy the cell membrane of *S. mutans*, resulting in the release of LDH from the cells (Richardson et al., 2008).

#### 3.5.2 Nucleic acid leakage of *S. mutans*

**Figure 7:**
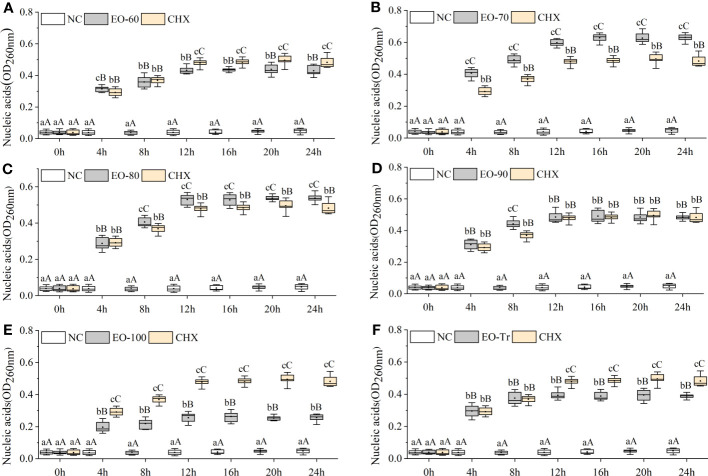
Effect of different ANEOs on nucleic acid leakage from *S. mutans*. The concentration of each ANEO is the MIC. **(A)** EO-60, **(B)** EO-70, **(C)** EO-80, **(D)** EO-90, **(E)** EO-100, **(F)** EO-Tr. Different lowercase letters represent statistically significant differences among the experimental time points in the same ANEO group (or NC) (*P* < 0.05), and different uppercase letters represent statistically extremely significant differences (*P* < 0.01). * represents statistically significant differences between the NC (or CHX) and the ANEO groups at the same time point (*P* < 0.05), ** represents statistically extremely significant differences (*P* < 0.01), and N represents no significant differences (*P* > 0.05).

Cell membrane is a kind of selectively permeable membrane. When the cell membrane is damaged, macromolecules such as nucleic acids leak out of the cell (Frallicciardi et al., 2022). The amount of nucleic acid leakage can be used for the determination and analysis of cell membrane integrity (Sun et al., 2018). As shown in **Figure 7B**, the OD_260_ of the EO-70 group rapidly increases to 0.628 ± 0.024 from 0 h to 16 h, and then stabilizes after 16 h (*P*< 0.05). From 0 to 12 h, the OD_260_ of other groups increased rapidly, and after 12 h the OD_260_ almost stopped increasing (*P*< 0.05) (**Figures 7A, C–F**). Compared with NC, the OD_260_ of all the ANEO groups was significantly higher 4 h later (*P*< 0.01). The OD_260_ of the EO-70, EO-80 and EO-90 groups was significantly higher than that of CHX after 4 h, 8 h, and 8 h respectively (*P*< 0.05) (**Figures 7B–D**). These results combined with the LDH leakage results indicated that all of the ANEOs can destroy the integrity of the cell membrane.

#### 3.5.3 Effects of ANEOs on cell membrane integrity

**Figure 8:**
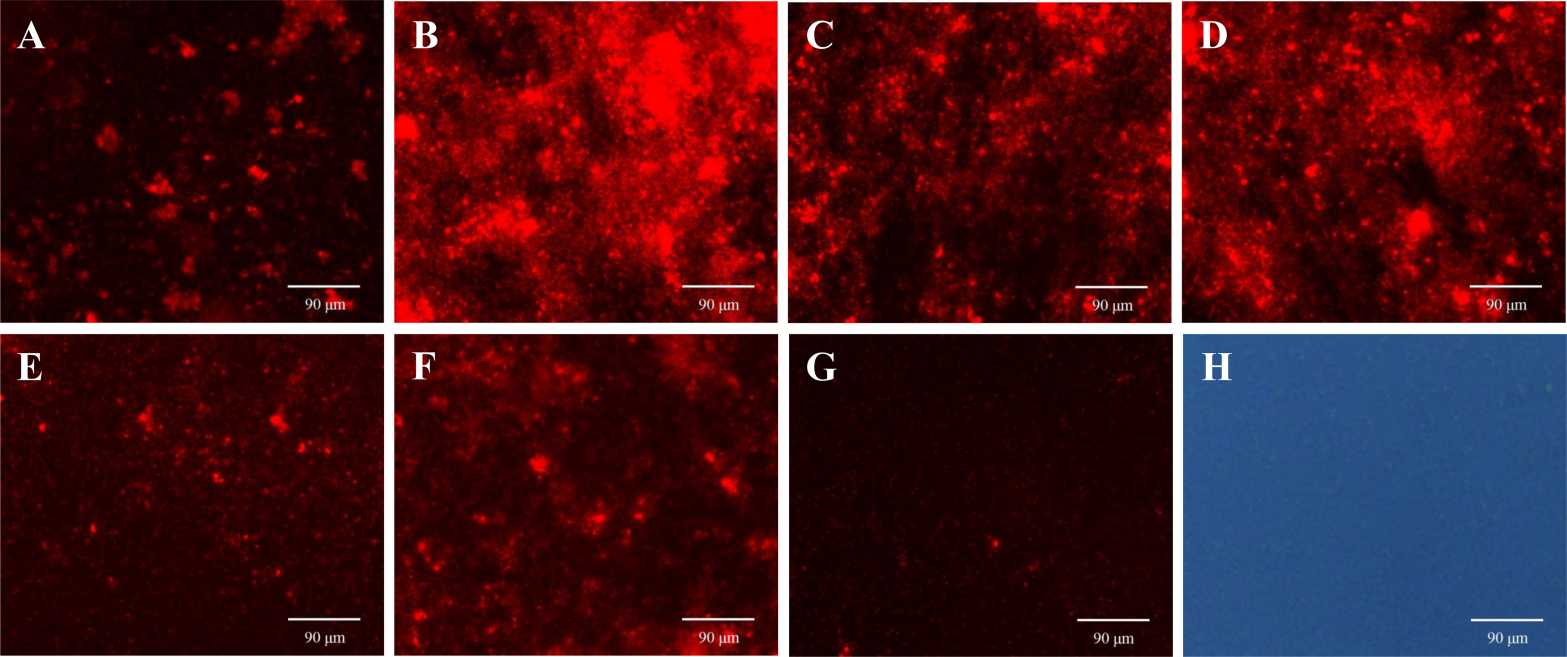
Effects of ANEOs on PI crossing the cell membrane of *S. mutans*. **(A)** EO-60, **(B)** EO-70, **(C)** EO-80, **(D)** EO-90, **(E)** EO-100, **(F)** EO-Tr, **(G)** Control (observed with a fluorescence microscope), **(H)** Control (observed with an optical microscope). Images were taken at ×40 objective magnification.

PI is a membrane impermeable and highly sensitive nucleic acid stain which releases a red fluorescence after the insertion of double-stranded DNA when cell membrane is damaged, and PI is usually used to evaluate the integrity of the cell membrane (Rosenberg et al., 2019). As shown in **Figure 8**, the red fluorescence intensity in the biofilms treated with ANEOs enhances compared with NC (**Figure 8G**). The fluorescence intensity in the EO-70 group were strongest (**Figure 8B**), followed by the EO-80 group and the EO-90 group (**Figures 8C, D**), while weaker fluorescence intensity appeared in the EO-60 and EO-TR groups (**Figures 8A, F**), and the EO-100 group showed the weakest (**Figure 8E**). Moreover, **Figures 8G, H** show that most cells in PBS are living cells, and PI is blocked outside the cell membrane of living cells, and displays weak fluorescence. Thus, the difference in the fluorescence intensity indicated not only that ANEOs could disrupt the structure of the bacterial cell membrane but also the strength of this ability was diverse for different ANEOs.

### 3.6 Effect of ANEOs on the morphology of *S. mutans*

**Figure 9:**
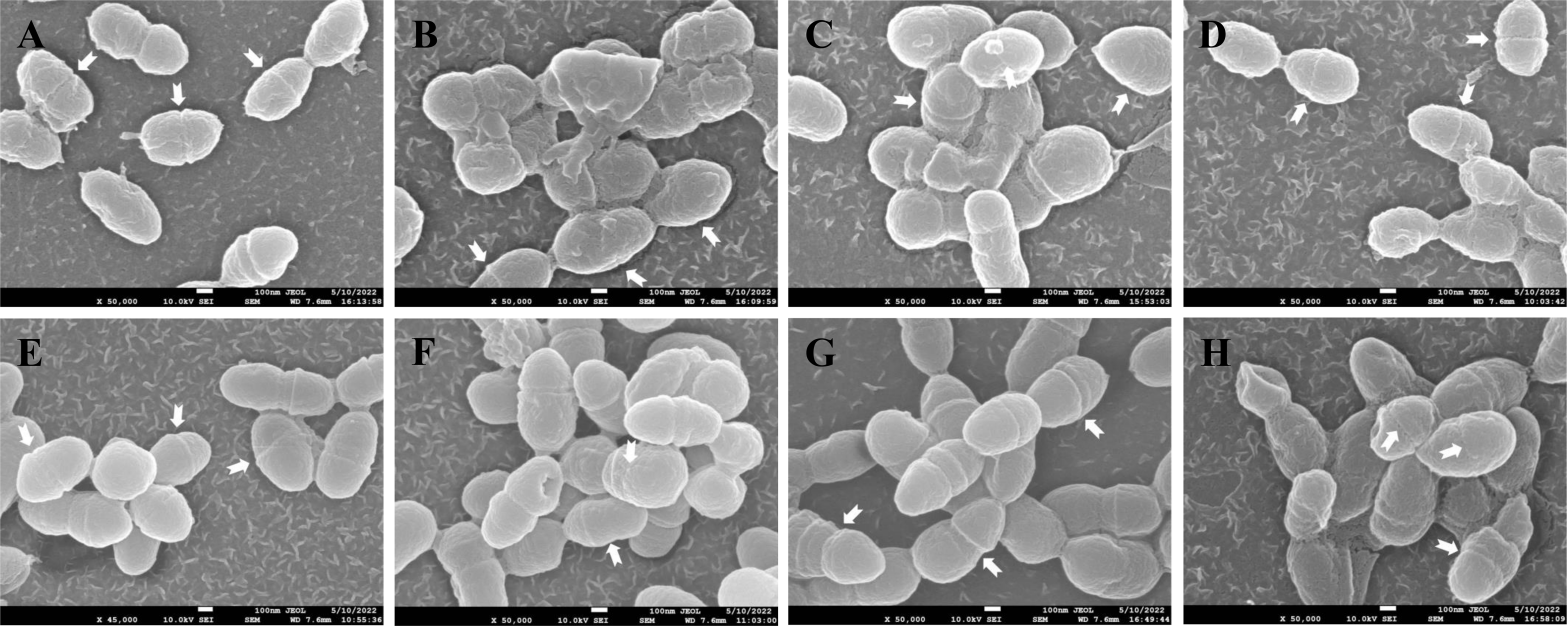
Effect of different ANEOs on the morphology of *S. mutans*. **(A)** EO-60, **(B)** EO-70, **(C)** EO-80, **(D)** EO-90, **(E)** EO-100, **(F)** EO-Tr, **(G)** NC (control), **(H)** CHX. Arrows point to division of the transverse groove.

SEM was used to analyze the damage of the *S. mutans* cell structure caused by ANEOs extracted at different temperatures. **Figure 9** shows that the structure of *S. mutans* is affected to varying degrees by the ANEOs, compared with NC. **Figure 9G** shows that the cells have a regular shape, smooth surface, an obvious transverse groove and are chain-like. After treatment with ANEOs and CHX, the bacteria cell surface was rough and even collapse, rupture (**Figures 9A–D, F, H**). The cells showed granules or granular accumulation (**Figures 9A, C, H**), and the cell chain was shortened or absent (**Figures 9B, D, E**). In addition, although no obvious division transverse groove was apparent, atrophy of the cells was evident, which suggests that cell division was blocked (**Figures 9A–E, H**). These results indicated that the cell morphology of *S. mutans* were damaged, and even the membrane system, which lead to the leakage of the cell contents. It is also apparent that ANEOs could interfere with normal cell division and affect the progress of the normal cell life cycle.

### 3.7 Cytotoxicity of ANEO in oral cells

To determine the safety of ANEOs, their cytotoxicity on HOECs was evaluated. The IC_10_ values of samples usually indicate non-cytotoxic concentrations (Attoff et al., 2017). As shown in **Table 1**, the IC_10_ values of six ANEOs to HOECs are higher than their MIC values, and the IC_10_ values of EO-70, EO-80 and EO-90 samples were even higher than their MBC values. The above data showed that the six ANEOs could inhibit the biofilm formation and damage the bacteria cell membrane of oral pathogenic bacteria, but had no cytotoxic effects on oral cells.

**Table 1:** IC_10_ values of ANEOs in HOECs.

Essential oil	IC_10_ values (mg/mL)
EO-60	1.10 ± 0.10
EO-70	3.16 ± 0.09
EO-80	2.19 ± 0.55
EO-90	3.39 ± 0.14
EO-100	2.81 ± 0.07
EO-Tr	1.94 ± 0.69

## 4 Discussion

Many infectious diseases in humans are caused or aggravated by biofilms, and dental caries is a typical biofilm-dependent disease (Koo et al., 2013; Horev et al., 2015). One important step in the caries occurrence is that pathogenic bacteria gather on the tooth surface and form a biofilm. Biofilms are highly organized bacterial colonies that are firmly attached to surfaces or interfaces and encased in a 3D extracellular polymeric matrix, such as EPS (Koo et al., 2013; Gao et al., 2016). *S. mutans* is the main contributor to dental caries biofilms, plays a significant role in the induction of dental caries because of its acidogenic, acid-tolerant and biofilm-forming characteristics (Yoo and Jwa, 2018), and is widely used as a target for dental caries research.

As shown in this study, the ANEOs displayed preferable antibacterial and antibiofilm activity against *S. mutans*. At MIC concentrations, all ANEOs could reduce the total biofilm biomass, and the total biofilm biomass of the EO-70 group was less than that of CHX. However, the total biofilm biomass could not entirely reflect the pathogenicity of the biofilm, and the inanimate bacteria in the biofilm may have lost pathogenicity. Thus, we analyzed the vitality of bacteria in the biofilm. The results showed that the metabolic activity of the biofilms treated by ANEOs was decreased, and a large number of bacteria were dead in the biofilm, further showing that the biofilm was less resistant to the ANEOs. In order to intuitively observe the damage of biofilm, we used an optimized biofilm model to observe its integrity. Frequently, to simulate the progression of dental caries, materials such as hydroxyapatite disks, bovine dentin disks or bovine teeth are used by researchers to construct dental caries biofilms (Ribeiro-Vidal et al., 2020; Neves et al., 2019; Vertuan et al., 2021). The biofilms formed in these models are relatively loose due to the hydrophobicity of material (Cui et al., 2018), and would not be suitable for analyzing the effects of ANEOs on cell arrangement in the biofilm. In this study, a polystyrene material more conducive to bacterial attachment was used to prepare biofilms. SEM images showed that ANEOs destroyed the dense structure of the biofilm, the layer becomes thinner, and the cell arrangement is irregular or loose (Xi et al., 2019). These data indicated that ANEOs decreased the total biomass and the metabolic activity, disrupted the integrity of the biofilm, reduced the cariogenic potential (Aiassa et al., 2016) and resistance to adverse conditions (i.e., antibiotics, high temperatures) of *S. mutans* biofilms (Muras et al., 2018).

In order to further explore the antibiofilm mechanism of the ANEOs, we analyzed their role in preventing biofilm formation. The dental biofilm life cycle generally includes the adhesion and colonization of bacteria, the assembly and maturation of 3D biofilms, biofilm degradation and re-colonization (Koo et al., 2013; Kumar et al., 2017). The adhesion and colonization of bacteria is the key factor determining whether the biofilm can be formed, and is the first step of biofilm formation. Surface hydrophobicity of bacteria directly affect their adhesion and colonization (Abdulla et al., 2014; Kozmos et al., 2021). ANEOs could reduce the surface hydrophobicity of *S. mutans*, weakening the adhesion and colonization of the bacteria, thus hindering the biofilm formation. EPS is a key component of dental biofilm formation and is also a virulence factor of cariogenic biofilms. (Lin et al., 2021). EPS provides a 3D scaffold for biofilm development, promotes local accumulation of bacteria on the teeth, and protects embedded bacteria (Bowen and Koo, 2011; Klein et al., 2015). The results of the EPS assay showed that every ANEO inhibited the production of WSP and WIP to varying degrees. In order to further explore the inhibition mechanism of ANEOs on EPS production, we analyzed the effect of ANEOs on the activity of the EPS synthetase, Gtf. The results of Gtf activity assay suggested that ANEOs inhibited Gtf activity. Therefore, we speculate that ANEOs prevented the synthesis of EPS by inhibiting the activity of Gtfs, and blocked 3D biofilm assembly, affected the maturity of biofilm and weakened the cariogenic potential of *S. mutans* (Kumar et al., 2017). In addition, EPS is the main material source of bacterial surface hydrophobicity (Devasia et al., 1993), the inhibition of EPS formation also reduced bacterial cell surface hydrophobicity, weakening the adhesion of the bacteria to surfaces.

The cell membrane is a type of selectively permeable membrane that prevents nucleic acids and other macromolecules from freely passing through it when it is in its normal state (Frallicciardi et al., 2022). If the cell membrane is damaged, the intracellular contents will leak; small molecules will flow out first, followed by RNA, DNA, proteins and other macromolecules. Thus, the leakage of intracellular substances such as RNA, DNA, and LDH is an important indicator to evaluate the integrity of the cell membrane (Sun et al., 2018). The results of LDH and nucleic acid leakage showed that ANEOs disrupted the integrity of the *S. mutans* cell membrane. In addition, the results of PI staining showed strong red fluorescence appearing in the ANEO groups, which indicated that PI had been inserted into the double-stranded DNA, and the cell membrane of *S. mutans* had been disrupted by the ANEOs. SEM images of *S. mutans* cells showed that ANEOs disrupted the cellular structure, leading to the disruption of the membrane system.

Based on results above, our study demonstrates that ANEOs have powerful antibacterial effect against dental caries pathogen *S. mutans*. Similarly, previous reports have shown that the extract of areca nut has antibacterial activity against bacterial and fungal strains, such as *Streptococcus, Staphylococcus aureus, Escherichia coli* and others (Peng et al., 2015). The volatile constituents extracted by Machová et al. exhibited best antibacterial effect against *Streptococcus* among 9 bacteria and yeast (Machová et al., 2021).

Some studies believe that chewing of areca nut can induce oral cancer and the arecoline is the main carcinogen (Oliveira et al., 2021). However, in 2020, the International Agency for Research on Cancer (IARC) reassessed the carcinogenicity of arecoline, and found that the evidence regarding cancer in humans for arecoline was “inadequate”, and no data were available (Marques et al., 2021). GC-MS data showed that there was no carcinogenic component arecoline in ANEOs. The MTT assay also showed that ANEOs had no cytotoxicity to normal oral cells. Therefore, the ANEO is safe as an oral antibacterial agent. GC-MS data also showed that the content of phenolic compounds in EO-70 was the highest, and the antibacterial activity of EO-70 was also the strongest. We speculate that phenolic compounds were the active antibacterial components of the ANEOs, which is consistent with reports that phenols generally have strong antibacterial activity (Fan et al., 2022), and the antibacterial components of areca nut are mainly polyphenol (Jam et al., 2021; Raju et al., 2021). Overall, the active components of ANEO were preliminarily separated and enriched through negative pressure hydro-distillation compared to the traditional hydro-distillation extraction. In addition, 36 more compounds were identified than traditional hydro-distillation extraction, and the low extraction temperature also can prevent the thermal degradation of essential oil components. Thus, this study sheds new light on the separation and enrichment of essential oil components. It is expected that the active components of essential oils can be precisely enriched and separated by improving the pressure regulation and temperature control elements to finely adjust the separation temperatures in future research.

## 5 Conclusion

This study successfully achieved preliminary separation of ANEO components by negative pressure hydro-distillation, providing some ideas for the separation of essential oil components of other plant. The obtained ANEOs showed good anti-bacterial and anti-biofilm activities against the main pathogenic bacteria of dental caries, *S. mutans*, by affecting the biofilm and disrupting the integrity of the cell membrane. These findings indicate that ANEOs might be a potential antibacterial agent for the prevention of dental caries.

## Data availability statement

The original contributions presented in the study are included in the article/**Supplementary Material**. Further inquiries can be directed to the corresponding authors.

## Ethics statement

Ethical approval was not required for the studies on humans in accordance with the local legislation and institutional requirements because only commercially available established cell lines were used.

## Author contributions

SL made substantial contributions to the design, the acquisition, analysis, and interpretation of data for the work. TZ made contributions to design of methodology, formal analysis, review, and editing. YW revised and edited it critically for important intellectual content and editing. ZL made contributions to design of methodology and formal analysis. LL revised it critically for important intellectual content. ZS made substantial contributions to the conception. All authors contributed to the article and approved the submitted version.
