# Supplementary material for: Antibacterial mechanism of areca nut essential oils against Streptococcus mutans by targeting the biofilm and the cell membrane
Source: Front Cell Infect Microbiol. 2023 Aug 28;13:1140689. doi: 10.3389/fcimb.2023.1140689 (PMC10494717; doi:10.3389/fcimb.2023.1140689)
Supplement: Supplementary file 1 [file DataSheet_1.docx]

Supplementary Material

Antibacterial mechanism of areca nut essential oils against *Streptococcus mutans* by targeting the biofilm and the cell membrane

# Supplementary Material and Methods

**S1: Negative pressure extraction device**

In order to explore the separation method of essential oil, preliminary separate different components of essential oil, and analyze the activity of different components of essential oil, a special negative pressure extraction device was assembled. Based on the principle that negative pressure can reduce the boiling point of solvent, substances with different volatilities are separated and collected according to the temperature from low to high. Under negative pressure, the sampling port of the traditional extractor of essential oil cannot be completely sealed, and the liquid and the collected oil in the tube is disturbed and can’t be separated due to the incoming air from the sampling port.

To solve the problem, the negative pressure extraction device shown in supplementary figure S1 was designed. In this device, the lower end of essential oil collection tube is sealed to avoid air leakage. A switch was set at the top of reflux tube, and its functions are as follows. First, it will prevent violent change of the air flow by closing this switch before switching off the vacuum pump. Second, when the essential oil extractor is removed, closing this switch can prevent the liquid in the extractor from flowing back and forth, and the essential oil from mixing with water again. Third, the gas leaking into the extractor from this switch can directly enter the condenser together with the steam, and will not pass through the liquid, which will not cause the vibration of the liquid in the reflux tube. In addition, there is a buffer bottle between the extractor and the extraction flask, which can alleviate the violent boiling and reflux of extraction solvent caused by negative pressure. The pressure control switch is used to adjust the internal pressure of the device, then the extraction solvent can boil at the set temperature. Generally, the volatility of substances is different. The preliminarily separation of strong and weak essential components in essential oil can be achieved by this device. The advantages of the new negative pressure distillation device are listed as follows.

(1) The lower end of essential oil collection tube is sealed to prevent damage to the essential oil layering due to air leakage.

(2) A switch was set at the reflux tube. Before the negative pressure is relieved, closing this switch can prevent violent changes in airflow, thereby avoiding disturbance to essential oils in the tube.

(3) Closing the switch on the reflux tube can stabilize the liquid in the collection tube, and prevent the essential oil from mixing with water again.

(4) The switch was set at the top of the reflux tube, and the gas entering from the switch can directly reach the condenser to prevent airflow from passing through the reflux liquid and causing liquid movement in the reflux tube.

(5) The precise pressure control switch is the key component for the experiment, which can achieve the boil of extraction solution at a set temperature.

(6) The buffer bottle can alleviate the violent boiling and back suction of the extraction solvent caused by negative pressure extraction, thereby reducing the probability of essential oil being contaminated by the extraction solvent.

(7) This device can boil the liquid in the reaction flask at different set temperatures, thereby separating different components of the essential oil.

(8) The extraction temperature in this device is generally below 100℃, preventing the thermal degradation of the biological activity components of essential oils.

**S2: Gas chromatography-mass spectrometry (GC-MS) analysis of ANEOs**

The ANEOs were analyzed by the GC/MS referring to the relevant literature (Yue et al., 2019) with slight changes. The GC/MS system consisted of an Agilent 7890A GC and Agilent 5977B MS detector, equipped with a HP-5MS column (30 m × 250 μm × 0.25 μm). A total of 5 μL of the essential oil sample (diluted with n-hexane) was injected in splitless mode. The injection port temperature was 250 ℃, He (99,995 % purity) was the carrier gas with a flow rate of 1.0 mL∙min^-1^. The primary oven temperature was programmed at 60 °C for 1min, then 10 ℃∙min^-1^ to 100 ℃, maintained 1 min, then 4 ℃∙min^-1^ to 220 ℃, kept isothermal for 1 min, finally 10 °C∙min^-1^ to 280 ℃ and left at 280 ℃ for 3 min, total 46 min. The mass spectrometer condition was as follows: ionization energy 70 eV; ion source temperature 230℃; quadrupole temperature 150 ℃; solvent delay 4 min; the mass scanning range was 50–1050 m/z. Identification of the compounds was carried out by comparing retention indices (RI) using C7-C40 n-alkanes as reference points and mass spectra with NIST 17 library data.

- Linear temperature programmed retention index was calculated by the following formula: *RI*=100*n*+100（*t_x_-t_n_*）/（*t_n_*_+1_-*t_n_*）

where *RI* represent retention indices of the target compound X; *t_x_* represent retention time of the target compound X; *t_n_* and *t_n_*_+1_ represent retention time of the n-alkanes that the number of carbon atoms are n and n+1, respectively, and *t_n_*<*t_x_*<*t_n_*_+1_.

**S3: Similarity analysis**

The similarity of the ANEOs were analyzed with “Similarity Evaluation System for Chromatographic Fingerprint of Traditional Chinese Medicine 2004A” software. The chromatograms of samples should be introduced into the software in the form of AIA (*.cdf or .txt), which included the information of peak area and retention time. The correlation coefficient of samples was calculated based on the peak area and retention time for evaluating the similarities of different chromatograms (Qian et al., 2020).

# Supplementary Results

**2.1 Constituents in different ANEOs**

The similarity of the chromatograms of different ANEOs were displayed in supplementary table S1. Major chemical constituents of each ANEO were shown in supplementary table S2. A total of 75 compounds were identified from ANEOs by the GC–MS analysis. The types and quantities of compounds in ANEOs extracted at different temperatures were remarkably different. There are 32, 31, 41, 45, and 19 compounds in EO-60, EO-70, EO-80, EO-90, and EO-100 respectively. In addition, 39 compounds are identified from EO-Tr, all of which are included in the above 75 compounds.

# Supplementary Figures and Tables

## Supplementary Figures

**
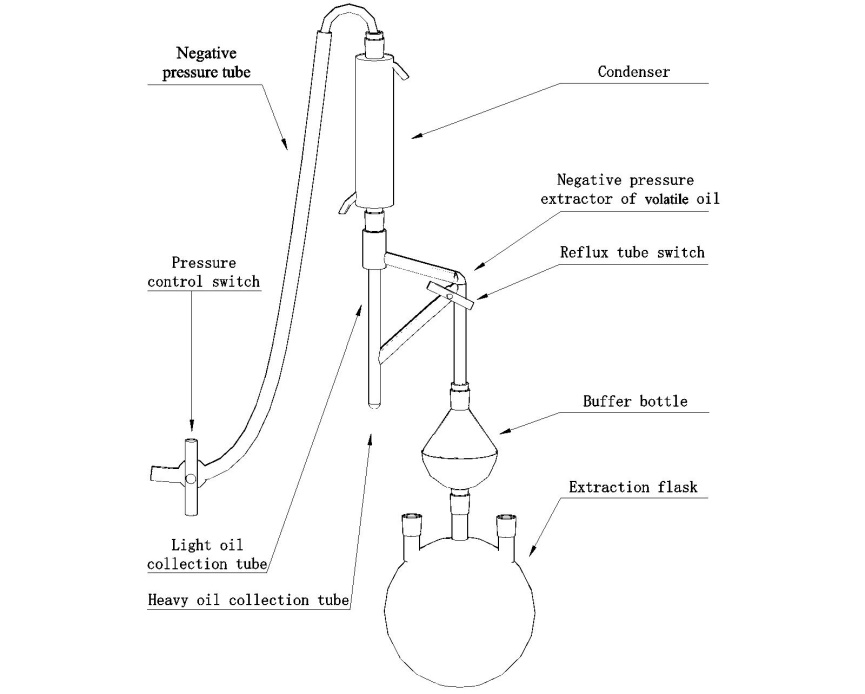
**

**Supplementary Figure S1.** Negative pressure extraction device of essential oil

## Supplementary Tables

**Supplementary Table S1.** The similarities of ANEOs extracted at different temperatures.

| Essential oil | EO-60 | EO-70 | EO-80 | EO-90 | EO-100 | EO-Tr |
| --- | --- | --- | --- | --- | --- | --- |
| EO-60 | 1.000 | 0.339 | 0.412 | 0.433 | 0.286 | 0.463 |
| EO-70 | 0.339 | 1.000 | 0.606 | 0.610 | 0.173 | 0.818 |
| EO-80 | 0.412 | 0.606 | 1.000 | 0.927 | 0.135 | 0.639 |
| EO-90 | 0.433 | 0.610 | 0.927 | 1.000 | 0.140 | 0.683 |
| EO-100 | 0.286 | 0.173 | 0.135 | 0.140 | 1.000 | 0.164 |
| EO-Tr | 0.463 | 0.818 | 0.639 | 0.683 | 0.164 | 1.000 |

**Supplementary Table S2.** Major chemical compositions of ANEOs.

| **Peak** | **Compounds** | **RI^a^** | **RT^b^** | **Area Pct /%** | | | | | |
| --- | --- | --- | --- | --- | --- | --- | --- | --- | --- |
|  |  |  |  | **EO-60** | **EO-70** | **EO-80** | **EO-90** | **EO-100** | **EO-Tr** |
| 1 | 2-methyl- Phenol | 1054.18 | 6.47 | - | 1.08 | 0.57 | 0.76 | - | 0.71 |
| 2 | 2-methoxy- Phenol | 1092.79 | 7.21 | - | 0.63 | 0.39 | 0.59 | - | 0.48 |
| 3 | 1,2-dimethoxy- Benzene | 1144.09 | 8.41 |  | 2.38 | 0.55 | 0.72 | - | 0.81 |
| 4 | 2,4-dimethyl- Phenol | 1151.56 | 8.59 | - | 2.94 | 1.82 | 2.32 | - | 2.35 |
| 5 | 3-ethyl- Phenol | 1171.08 | 9.06 | - | 0.85 | 0.71 | 0.91 | - | 1.12 |
| 6 | (R)-4-methyl-1-(1-methylethyl)-3-Cyclohexen-1-ol | 1180.63 | 9.29 | 1.01 | 2.29 | 0.99 | - | - | - |
| 7 | Terpinen-4-ol | 1181.04 | 9.30 | - | - | - | 1.14 |  | 1.76 |
| 8 | 2-Methoxy-5-methylphenol | 1189.76 | 9.51 | 0.87 | 0.79 | 0.34 | - | - | 0.49 |
| 9 | 2-Methoxy-4-Methyl Phenol | 1195.16 | 9.64 | - | 3.62 | 2.37 | 3.69 | 1.12 | 3.53 |
| 10 | 3,4-Dimethoxytoluene | 1238.99 | 10.82 | 2.98 | 12.88 | 6.93 | 7.18 | 2.51 | 6.63 |
| 11 | 2-ethyl-6-methyl- Phenol | 1244.86 | 10.98 | - | 1.79 | - | - | - | 1.58 |
| 12 | 2-isopropyl-5-methyl-3-Cyclohexen-1-one | 1256.96 | 11.31 | - | 1.40 | 0.95 | 0.73 | - | 0.92 |
| 13 | 2-ethyl-4-methyl- Phenol | 1262.83 | 11.47 | - | 1.81 | - | - | - | - |
| 14 | p-Cumenol | 1264.66 | 11.52 | - | - | - | 1.39 | - | - |
| 15 | 4-ethyl-2-methoxy-phenol | 1281.16 | 11.97 | 2.12 | 10.61 | 7.21 | 8.55 | 1.97 | 8.42 |
| 16 | (Z)-1-methoxy-4-(1-propenyl)- Benzene | 1287.40 | 12.14 | - | 2.88 | - | - | - | 2.64 |
| 17 | cis-Anethol | 1288.50 | 12.17 | - | - | 1.55 | 1.47 | - | - |
| 18 | 2,3-dihydro-2-methyl-1H-Inden-1-one | 1308.87 | 12.74 | - | - | 0.57 | 0.51 | - | - |
| 19 | 1,2,3-Trimethoxybenzene | 1313.06 | 12.86 | - | 1.04 | 0.90 | 1.17 | - | 1.69 |
| 20 | 1-Methylindan-2-one | 1318.56 | 13.02 | - | 0.76 | - | - | - | - |
| 21 | p-tert-Butylcatechol | 1322.72 | 13.14 | 2.66 | - | - | - | 1.89 | - |
| 22 | 1-(2-hydroxy-5-methoxyphenyl)- Ethanone | 1323.75 | 13.17 | - | 8.78 | - | - | - | 6.15 |
| 23 | 5-methoxy-2,3,4-trimethyl- Phenol | 1325.14 | 13.2**1** | - | - | 5.70 | 4.70 | - | - |
| 24 | 4-(1-methylpropyl)- Phenol | 1333.10 | 13.44 | - | - | 1.60 | - | - | - |
| 25 | Coumarin | 1350.75 | 13.95 | - | 2.02 | - | - | - | - |
| 26 | 3-Allyl-6-methoxyphenol | 1359.06 | 14.19 | 0.69 | 4.44 | - | - | - | - |
| 27 | Eugenol | 1359.75 | 14.21 | - | - | 3.49 | 3.44 | - | 3.34 |
| 28 | 2-methoxy-4-propyl- Phenol | 1369.10 | 14.48 | 3.86 | 7.18 | 5.77 | 4.09 | 1.96 | 4.93 |
| 29 | 2,5-Dihydroxypropiophenone | 1375.67 | 14.67 | - | - | - | 1.23 | - | - |
| 30 | 7-Methylindan-1-one | 1392.98 | 15.17 | - | - | - | 0.74 | - | - |
| 31 | 7-methyl-Bicyclo[4.2.0]octa-1,3,5-triene | 1393.33 | 15.18 | - | - | 0.71 | - | - | 0.41 |
| 32 | Methyleugenol | 1406.44 | 15.56 | 1.65 | 4.35 | 4.92 | 4.4 | 1.73 | 6.39 |
| 33 | 1,2-Dimethoxy-4-n-propylbenzene | 1409.53 | 15.65 | 3.26 | 4.94 | 4.26 | 2.93 | 2.07 | 5.00 |
| 34 | 6-ethyl-1,2,3,4-tetrahydro- Naphthalene | 1423.63 | 16.06 | - | - | - | 0.47 | - | - |
| 35 | 1-(1,1-dimethylethyl)-4-ethenyl- Benzene, | 1433.93 | 16.36 | - | - | 2.98 | - | - | - |
| 36 | 2-methoxy- Naphthalene | 1449.41 | 16.81 | 1.34 | 2.67 | 2.48 | 1.93 | - | 2.34 |
| 37 | 2,2'-methylenebis(5-methyl- Furan) | 1468.32 | 17.36 | - | 2.23 | - | - | - | - |
| 38 | 2'-Hydroxy-3',4'-dimethoxyacetophenone | 1480.70 | 17.72 | 1.58 | 3.43 | - | - | 1.66 | 4.65 |
| 39 | 3,4,5-trimethoxy- Benzaldehyde | 1481.39 | 17.74 | - | - | 4.23 | 4.08 | - | - |
| 40 | 4-butyl-1,2-dimethoxy- Benzene | 1505.94 | 18.45 | - | 0.94 | 1.67 | 1.28 | - | 1.10 |
| 41 | Dibenzofuran | 1512.62 | 18.64 | 1.27 | 1.26 | - | 1.14 |  | 1.26 |
| 42 | 3,5-Dimethoxybenzyl alcohol | 1514.03 | 18.68 | - | *-* | 1.35 | *-* | *-* | *-* |
| 43 | methyl ester 10-methyl- Undecanoic acid | 1521.77 | 18.90 | 2.29 | - | - | - | - | - |
| 44 | 1,2,3-trimethoxy-5-(2-propenyl)- Benzene | 1556.61 | 19.89 | - | - | - | 1.10 | - | 0.72 |
| 45 | 2-Methoxy-4-nitrophenyl isothiocyanate | 1559.78 | 19.98 | - |  | - | - | 2.24 | - |
| 46 | 1-(4-hydroxy-3,5-dimethoxyphenyl)-1-Propanone | 1561.19 | 20.02 | - | - | 3.69 |  | - | - |
| 47 | Dodecanoic acid | 1572.80 | 20.35 | 7.48 | - | 2.52 | 1.40 | 8.87 | 3.05 |
| 48 | Fluorene | 1578.78 | 20.52 | 2.84 | 1.95 | 1.76 | 3.35 | 1.05 | 1.36 |
| 49 | [1,1'-Biphenyl]-4-carboxaldehyde | 1617.75 | 21.61 | - | - | 0.7 | - | - | - |
| 50 | 4-methyl- Dibenzofuran | 1630.52 | 21.96 | 1.88 | 0.78 | 1.01 | 0.83 | - | 0.53 |
| 51 | 4-methoxy-1,1'-Biphenyl | 1655.31 | 22.64 | - | - | 1.38 | 0.71 | - | 0.54 |
| 52 | 2-Hydroxyfluorene | 1662.60 | 22.84 | 2.13 | - | 1.21 | 0.78 | - | - |
| 53 | 1-methyl-9H-Fluorene | 1692.86 | 23.67 | 5.43 | 1.27 | - | - | 1.47 | 0.70 |
| 54 | 2-methyl-9H-Fluorene | 1693.59 | 23.69 | 1.60 | - | 2.69 | 2.44 | - | - |
| 55 | Methyl tetradecanoate | 1722.18 | 24.45 | 1.74 | - | - | - | - | - |
| 56 | Tetradecanoic acid | 1765.08 | 25.58 | 3.77 | - | 0.68 | 1.89 | - | 2.55 |
| 57 | Anthracene | 1772.68 | 25.78 | 1.67 | - | 0.52 | 0.54 | 14.21 | 0.36 |
| 58 | Phenanthrene | 1773.44 | 25.80 | 7.08 | 1.65 | 2.03 | 1.88 | - | 2.00 |
| 59 | Methyl 9-methyltetradecanoate | 1840.03 | 27.51 | 1.00 | - | - | - | - | - |
| 60 | 6,10,14-trimethyl-2-Pentadecanone | 1841.22 | 27.54 | 2.31 | - | 0.32 | 0.80 | 2.61 | 0.72 |
| 61 | Pentadecanoic acid | 1862.22 | 28.07 | - | - | - | 0.51 | 2.73 | 0.63 |
| 62 | 2-methyl- Anthracene | 1886.78 | 28.69 | 1.32 | - | 0.19 | - | - | - |
| 63 | 2-methyl- Phenanthrene | 1892.33 | 28.83 | 2.43 | - | 0.23 | 0.99 | - | - |
| 64 | 3-methyl- Phenanthrene | 1900.26 | 29.03 | 1.06 | - | - | - | - | - |
| 65 | methyl ester 14-methyl-Pentadecanoic acid | 1922.61 | 29.57 | 7.16 | - | - | - | - | - |
| 66 | Palmitoleic acid | 1941.64 | 30.03 | - | - | - | - | 2.27 | 0.47 |
| 67 | n-Hexadecanoic acid | 1976.40 | 30.87 | 4.44 | - | 0.52 | 2.47 | 32.86 | 5.92 |
| 68 | Fluoranthene | 2049.36 | 32.58 | - | - | - | 0.15 |  |  |
| 69 | methyl ester 10,13-Octadecadienoic acid | 2088.77 | 33.49 | - | - | - | 0.19 | - | - |
| 70 | (E,E)-methyl ester 9,12-Octadecadienoic acid | 2090.50 | 33.53 | 6.22 | - | - | - | - | - |
| 71 | methyl ester 11-Octadecenoic acid | 2095.27 | 33.64 | - | - | - | 0.17 | - | - |
| 72 | (Z,Z,Z)-methyl ester,9,12,15-Octadecatrienoic acid | 2097.00 | 33.68 | 4.87 | - | - | - | - | - |
| 73 | Linoelaidic acid | 2132.45 | 34.47 | - | - | - | - | - | 1.4 |
| 74 | (Z,Z)-9,12-Octadecadienoic acid | 2139.20 | 34.62 | - | - | - | 0.31 | 10.47 | - |
| 75 | 10(E),12(Z)-Conjugated linoleic acid | 2143.70 | 34.72 | - | - | - | 0.30 | 4.14 | 0.73 |

^a^ RI, retention index relative to n-alkane C_7_-C_40_ on the HP-5MS column.

^b^ RT, retention time.

**References**

Yue, Y., Zhang, Q., and Wang, J. (2019). Integrated gas chromatograph-mass spectrometry (GC/MS) and MS/MS-based molecular networking reveals the analgesic and anti-inflammatory phenotypes of the sea slater Ligia exotica. Marine drugs, 17(7), 395.

Qian, Z., Yiyang, C., Lixia, M., Yue, J., Jun, C., Jie, D., et al. (2020). Study on the Fingerprints and Quality Evaluation of Angelica Sinensis Radix by HPLC Coupled with Chemometrics Based on Traditional Decoction Process of ACPTCM. Dose-Response, 18(3), 1559325820951730.
